# Pre-diagnostic prognostic value of leukocytes count and neutrophil-to-lymphocyte ratio in patients who develop colorectal cancer

**DOI:** 10.3389/fonc.2023.1148197

**Published:** 2023-06-05

**Authors:** Giulia Turri, Simone Caligola, Stefano Ugel, Cristian Conti, Silvia Zenuni, Valeria Barresi, Andrea Ruzzenente, Giuseppe Lippi, Aldo Scarpa, Vincenzo Bronte, Alfredo Guglielmi, Corrado Pedrazzani

**Affiliations:** ^1^ Division of General and Hepatobiliary Surgery, Department of Surgical Sciences, Dentistry, Gynecology and Pediatrics, University of Verona, Verona, Italy; ^2^ Istituto Oncologico Veneto IRCCS, Padova, Italy; ^3^ Immunology Section, University Hospital and Department of Medicine, University of Verona, Verona, Italy; ^4^ Department of Diagnostics and Public Health, Section of Pathology, University of Verona, Verona, Italy; ^5^ Department of Neurological, Biomedical and Movement Sciences, Section of Clinical Biochemistry, University of Verona, Verona, Italy

**Keywords:** colorectal cancer, inflammatory markers, prognosis, diagnosis, leucocytes, inflammation

## Abstract

**Introduction:**

Emerging evidence is pointing towards a relevant role of immunity in cancer development. Alterations in leukocytes count and neutrophil-to-lymphocyte ratio (NLR) at diagnosis of colorectal cancer (CRC) seems to predict poor prognosis, but no data is available for the pre-diagnostic values.

**Methods:**

Retrospective analysis of patients who underwent surgery for CRC at our center (2005 – 2020). 334 patients with a complete blood count dating at least 24 months prior to diagnosis were included. Changes in pre-diagnosis values of leukocytes (Pre-Leu), lymphocytes (Pre-Lymph), neutrophils (Pre-Neut), and NLR (Pre-NLR) and their correlation with overall- (OS) and cancer-related survival (CRS) were analyzed.

**Results:**

Pre-Leu, Pre-Neut and Pre-NLR showed an increasing trend approaching the date of diagnosis, while Pre-Lymph tended to decrease. The parameters were tested for associations with survival after surgery through multivariable analysis. After adjusting for potential confounding factors, Pre-Leu, Pre-Neut, Pre-Lymph and Pre-NLR resulted independent prognostic factors for OS and CRS. On sub-group analysis considering the interval between blood sampling and surgery, higher Pre-Leu, Pre-Neut, and Pre-NLR and lower Pre-Lymph were associated with worse CRS, and the effect was more evident when blood samples were closer to surgery.

**Conclusion:**

To our knowledge, this is the first study showing a significant correlation between pre-diagnosis immune profile and prognosis in CRC.

## 1 Introduction

The relationship between inflammation and cancer has been widely investigated. As early as in the 19^th^ century, mounting evidence suggested a correlation between inflammatory processes and cancer development (1, 2). Epidemiological studies confirmed that chronic inflammation predisposes to several forms of cancer, including colorectal carcinoma (CRC) in patients with inflammatory bowel diseases (1). More recently, immunotherapy has shown promising results as an effective treatment in patients with various cancers, especially in those with melanoma or breast cancer (3, 4), but some evidence of its potential efficacy has also been found in subjects with CRC (5, 6).

The immunity-cancer relationship is most likely bi-directional. On one hand, prolonged inflammatory stigmata trigger cancer cells proliferation and angiogenesis, thus facilitating genetic mutations and the acquisition of a malignant profile (2, 7) as well as metastatic spread (7). On the other hand, cancer cells may secrete themselves pro-inflammatory cytokines able to recruit immune cells in the tumor micro-environment (TME) (8). Immune cells composing TME could play a facilitating or an antitumor response, depending on the involved subset of cells (9). Recent studies suggested that cancer progression may result from imbalances between tumor growth and the host immune surveillance (10, 11). Interestingly, several studies have found a relevant association between survival of patients with different cancers and alterations in peripheral immune cells count (12–15). The latter may in fact play a role as circulating biomarkers of an immune shift leading to tumor escape (16). Particular attention has been directed towards cellular response, identifying neutrophils, lymphocytes, leucocytes, monocytes, platelets, and some of their ratios as main prognostic factors in CRC (17–21). Previous analyses evaluated exclusively pre-operative values of circulating cells, but changes in immune profile may take place months or years before cancer diagnosis given its implication in tumor etiopathogenesis (22). Only few studies assessed longitudinal changes of pre-diagnosis systemic inflammatory markers in cancer patients (22–25), but no data are available for CRC.

The aim of this retrospective study was to evaluate the pre-diagnostic trend and the prognostic significance of pre-diagnosis leukocyte count and neutrophil-to-lymphocyte ratio (NLR) in patients who develop CRC.

## 2 Materials and methods

### 2.1 Inclusion criteria

The initial study population consisted of patients who underwent surgery for CRC at the Division of General and Hepatobiliary Surgery, University of Verona Hospital Trust between January 2005 and December 2020. The clinical data were retrieved from a retrospective single-center database. Age of 18 years or older, urgent or elective surgery, histological diagnosis of CRC, clinical-pathological and follow-up data, and availability of a complete blood count dated at least 24 months before surgery were the inclusion criteria of this study.

### 2.2 Preoperative work-up and histopathological staging

All elective patients underwent preoperative staging by colonoscopy, thoracoabdominal CT scan and tumor markers (CEA, CA 19-9). Additional diagnostic modalities (e.g. MRI, PET-CT) were used when clinically indicated. The preoperative assessment of urgent cases varied depending on clinical necessities. All patients underwent preoperative routine laboratory tests.

The specimens were examined through routine histopathological analysis. Tumor staging was assessed according to the 8^th^ Edition of the American Joint Committee on Cancer (AJCC) and the Union International Contre Le Cancer (UICC) (26). The presence of residual tumour after resection was described using the AJCC/UICC terminology (R classification). Patients were grouped according to the presence (R2) or absence (R0-R1) of macroscopic residual tumour.

### 2.3 Assessment of pre-diagnosis blood tests

Data regarding pre-diagnosis blood tests were obtained by querying the Medical Laboratory database system into which all laboratory results were stored. Blood samples associated with admission to the Emergency Department or with clear evidence of an ongoing infection were excluded. Patients with autoimmune disorders and receiving corticosteroids or immunosuppressive/immunomodulatory treatments were also excluded. For each patient operated on within the inclusion period, one complete blood count (CBC) with differential dated at least 24 months before surgery was obtained. When more than one blood test was available, we recorded the closer to diagnosis. Pre-neutrophil-to-lymphocyte ratio (Pre-NLR) was calculated by dividing the absolute number of Pre-neutrophils (Pre-Neut) by the absolute number of Pre-lymphocytes (Pre-Lymph). The CBC was performed using Advia 2120 (Siemens Healthcare Diagnostics, Tarrytown NY, USA). The local reference ranges are 4.3– 10.0 × 10^9^/L for total leucocytes, 2.0–7.0 × 10^9^/L for neutrophils, and 0.95–4.5 × 10^9^/L for lymphocytes. The same analyser was used throughout the study period, and the quality and reproducibility of test results was validated by data of both internal quality control and external quality assessment.

### 2.4 Extent of surgery

The main goal of surgery was the complete removal of the tumor (R0 resection), although palliative surgery was carried out in selected cases to treat tumor-related complications. Extent of surgery was planned considering patient’s performance status and age and primary tumour location. Surgical approach (open versus minimally invasive) was based on surgeon’s preference and expertise. Anatomical resections with ligation of vessels at their origin were the procedures of choice in order to achieve an adequate lymphadenectomy (27).

### 2.5 Data collection and follow-up

Clinical and pathological data were retrieved from a retrospective database. The analyzed variables included demographic, clinical, surgical, and pathological characteristics. Patients who died within 30 days from surgery were not considered for survival analysis. Survival and follow-up data were obtained by collecting outpatient clinical records or by contacting the patients or their relatives. Data about recurrence, status at most recent follow-up, and cause of death were registered. Overall-survival (OS) was defined as the length of time between surgery and death from any cause. Cancer-related survival (CRS) was measured from the date of surgery to the date of death from CRC, whilst patients who died from causes other than cancer were considered censored at the time of death. All methods used in this study were performed in accordance with the relevant ethical guidelines and regulations of the University Hospital of Verona, where the investigation was carried out. Informed consent was obtained from all patients, and the study protocol was approved by the local ethical committee (Comitato Etico per la Sperimentazione Clinica delle Province di Verona e Rovigo, Azienda Ospedaliera Universitaria Integrata Verona; ID number: 58642-CRINF-1560CESC).

### 2.6 Statistical analysis

Continuous data were presented as means ± standard deviations (SD) or medians (ranges), as appropriate. Categorical data were presented as frequencies. All parameters of Pre-CBC were tested for potential associations with survival after surgery, and those with adequate numerosity and significant associations are presented in the results (Pre-leukocytes (Pre-Leu), Pre-Neut, Pre-Lymph, Pre-NLR). Multivariate analysis of OS and CRS was performed using Cox regression. We fitted an individual Cox regression model for Pre-Leu, Pre-Neut, Pre-Lymph and Pre-NLR adjusting for the following covariates: age, gender (female vs. male), tumor location (rectum vs. colon), histological type (mucinous vs. non-mucinous), setting of surgery (elective vs. urgent), and AJCC/UICC TNM stage (stage II, stage III and stage IV vs. stage I). Pre-Leu, Pre-Neut, Pre-Lymph, Pre-NLR and age were treated as continuous variables. Prior to fitting the Cox regression model, the distribution of the continuous parameters was inspected and patients with outlier values were removed from the analysis after initial inclusion. Martingale residuals were used to assess the functional form of the continuous covariates and to assess the overall fit of the Cox model. Nonlinearity of the parameters was addressed using spline terms. To choose the model, the goodness-of-fit was assessed using the likelihood ratio test between models with or without spline terms for the continuous parameters. Pre-Leu, Pre-Neut, Pre-NLR and age were treated as simple linear covariates while Pre-Lymph were modeled with a linear spline with a single knot located in the median. The proportional hazards assumption was checked using the Schoenfeld residual-based test. The variable ‘intent of surgery (R0, R1, R2)’ was stratified in each Cox model because it violated the proportional hazard assumption. Multicollinearity was checked computing the variance inflation factors (VIF). The significance of the parameters was assessed through the analysis of variance (ANOVA) considering a p-value < 0.05 statistically significant. Hazard ratios (HR) for continuous parameters were computed as interquartile range (IQR) effects. A subsequent analysis was conducted considering the interval between CBC sample and surgery. The variable ranged between 24 and 180 months, therefore it was sub-categorized into 4 groups: 24-36 months, 37-60 months, 61-120 months, and >120 months before surgery. Survival curves were built estimating the survival probability of the patients in the different time intervals before surgery and considering the 1^st^ quartile, median and 3^rd^ quartile of Pre-Leu, Pre-Neut, Pre-Lymph and Pre-NLR. To accomplish this task, the parameter ‘months before surgery’ was included in the Cox model for this analysis. The statistical analysis was performed with the R (v4.1.0) programming language and functions of the ‘rms’ and ‘survival’ (v3.2-11) packages. The figures were generated using the R packages ‘rms’, ‘ggplot2’ (v3.3.3), ‘ggpubr’ (v0.4.0), ‘MASS’ and ‘scales’.

## 3 Results

### 3.1 Patients under study

During the study period, 1674 patients underwent urgent and elective surgery for CRC. Among these, we were able to retrieve pre-diagnosis full blood count values for 334 patients. The median interval (interquartile range, IQR) between the pre-diagnosis blood sample and surgery was 50 (36 – 84) months.

**Table 1** describes the clinic-pathological characteristics of the cohort under study. Median age (range) at diagnosis was 70.1 (27.9 – 91.6) years. Most of the CRC were localized in the right colon (*n* = 135, 40.4%) followed by the rectum (*n* = 129, 38.6%). Stages were almost equally distributed within the cohort. Most patients underwent potentially curative surgery (R0 *n* = 290, 86.8%; R1 *n* = 8, 2.4%). 72 patients (21.6%) presented mucinous histology. **Table 1** also presents the mean values of pre-diagnosis parameters, which were all within normal ranges.

**Table 1:** Characteristics of the cohort under study.

	Patients	Data
Age, median (range)	334	70.1 (27.9 – 91.6)
Gender	334	
Male		204 (61.1)
Female		130 (38.9)
CEA, median (range)	195	2.6 (0.1 – 1276.8)
Tumor location	334	
Right colon		135 (40.4)
Left colon		70 (21.0)
Rectum		129 (38.6)
Type of surgery, elective	334	313 (93.7)
Intent of surgery, curative (R0-1)	334	298 (89.2)
Pathological TNM stage	334	
I		96 (28.7)
II		98 (29.3)
III		84 (25.1)
IV		56 (16.8)
Histology, adenocarcinoma	334	262/78.4)
Grading, poorly differentiated (G3)	262	34 (10.2)
Pre-diagnosis complete blood count		
Hb (g/dL), mean (SD)	334	14.2 (1.7)
Platelets, mean (SD)	334	253.9 (75.8)
RDW, mean (SD)	334	13.6 (1.2)
Leukocytes, mean (SD)	334	7.2 (1.9)
Neutrophils, mean (SD)	334	4.2 (1.6)
Lymphocytes, mean (SD)	334	2.1 (0.7)
NLR, mean (SD)	334	2.3 (1.3)

Data in parentheses are percentages unless specified otherwise.

SD: standard deviation; NLR: neutrophils-to-lymphocytes ratio.

### 3.2 Longitudinal analysis of Pre-CBC

**Figure 1:**
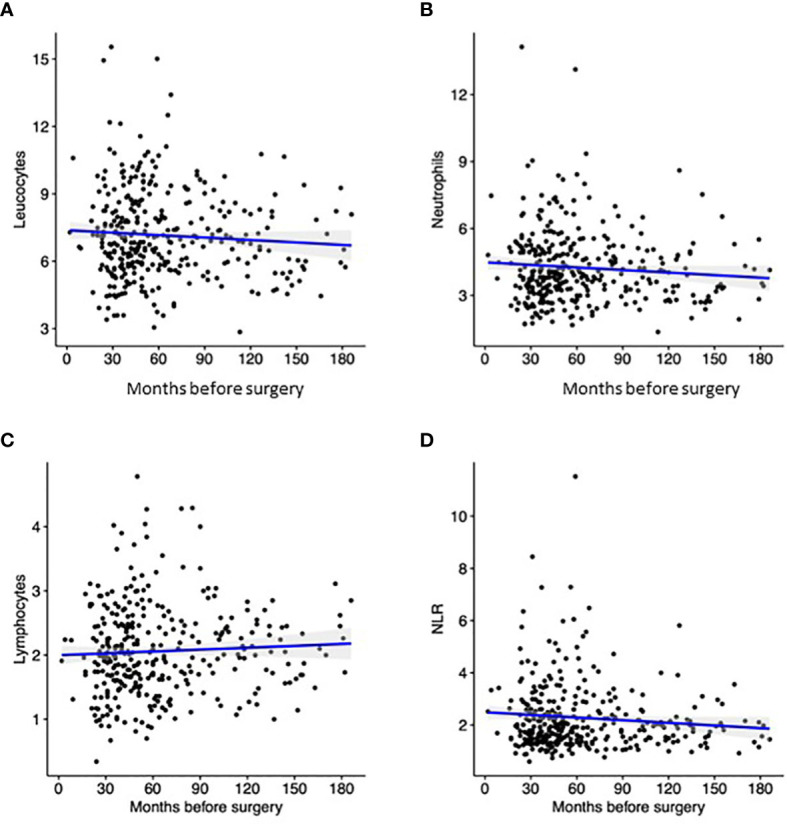
Distribution Pre-Leu **(A)**, Pre-Neut **(B)**, Pre-Lymph **(C)**, and Pre-NLR **(D)** according to the interval between blood sampling and surgery. Black dots correspond to the discrete value of each patient, while the blue line represent the trend according to linear regression.

Being a retrospective study, the interval between Pre-CBC and surgery varied in our cohort, from a minimum of 24 months to a maximum of 180 months. Temporal distribution of parameters is depicted in **Figures 1A–D**. Despite the correlation index was quite low for all parameters, it should be noted that the value of Pre-Leu, Pre-Neut and Pre-NLR tended to be higher close to surgery, while the value of Pre-Lymph showed an opposite trend.

### 3.3 Association between Pre-CBC and survival

All parameters of Pre-CBC were tested for potential associations with survival after surgery through multivariable analysis. An individual Cox regression was conducted for Pre-Leu, Pre-Neut, Pre-Lymph and Pre-NLR adjusting for age, gender, tumor location, histological type, setting of surgery, and AJCC/UICC pTNM stage as previously described. As shown in **Table 2**, all the parameters of Pre-CBC resulted to be independent prognostic factors for OS and CRS. Other significant prognostic factors were age for OS (p<0.001), and TNM stage for both OS and CRS (p<0.001).

**Table 2:** Multivariable analysis for OS and CRS.

	OS HR (95% CI)	p value	CRS HR (95% CI)	p value
Age	2.03 (1.47 – 2.79)	**<0.001**	1.36 (0.92 – 2.01)	0.13
Gender		0.99		0.49
Male	–		–	
Female	1.00 (0.67 – 1.48)		1.20 (0.71 – 2.00)	
Tumor location		0.47		0.56
Colon	–		–	
Rectum	0.83 (0.50 – 1.38)		0.83 (0.44 – 1.57)	
Type of surgery		0.07		0.24
Elective	–			
Urgent	1.81 (0.95 – 3.47)		1.77 (0.68 – 4.56)	
Pathological TNM stage		**<0.001**		**<0.001**
I	–		–	
II	1.68 (0.87 – 3.25)		0.91 (0.32 – 2.59)	
III	2.88 (1.51 – 5.50)		3.68 (1.52 – 8.89)	
IV	6.1 (2.89 – 12.87)		9.18 (3.51 – 24.06)	
Histological type		0.10		0.36
Adenocarcinoma	–		–	
Mucinous	0.61 (0.36 – 1.04)		0.68 (0.35 – 1.32)	
Pre-NLR*	1.41 (1.22 – 1.62)	**<0.001**	1.51 (1.26 – 1.81)	**<0.001**
Pre-Leukocytes*	1.37 (1.09 – 1.71)	**0.006**	1.61 (1.121 – 2.13)	**<0.001**
Pre-Neutrophils*	1.41 (1.17 – 1.70)	**0.003**	1.65 (1.30 – 2.09)	**<0.001**
Pre-Lymphocytes*	0.76 (0.62 – 0.94)	**<0.001**	0.76 (0.59 – 0.99)	**<0.001**

*Hematological parameters were inputted separately in multivariable analysis. We report HR (95% CI) of the multivariable analysis including NLR.

Statically significant p values are reported in bold.

"-" refers to the reference category.

**Figure 2:**
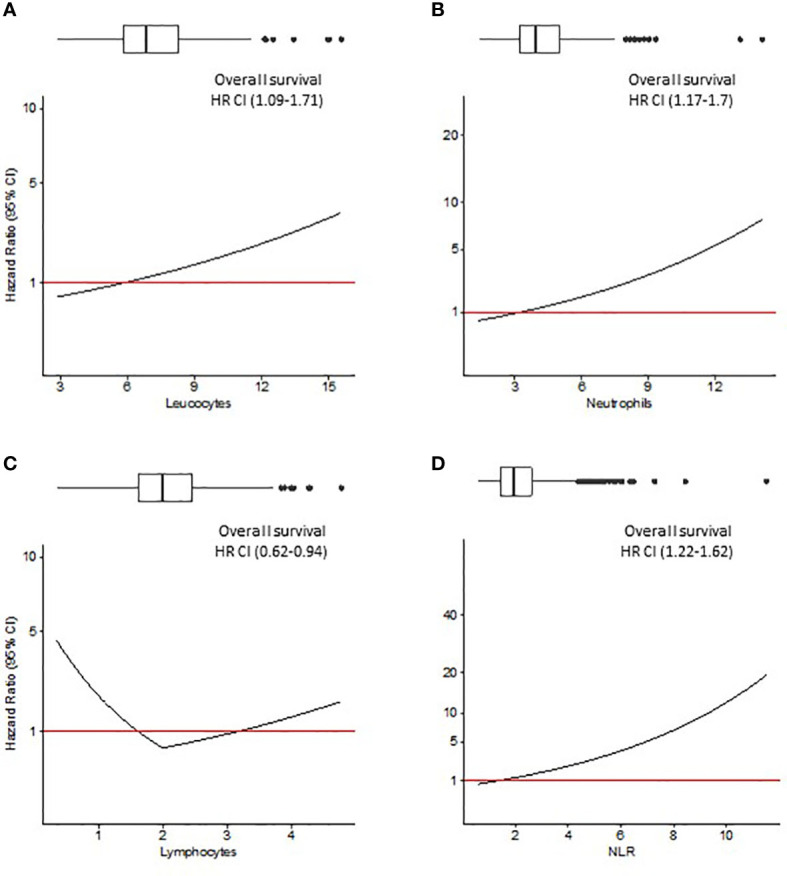
Overall survival (OS) analysis. Hazard ratio (HR) resulting from Cox regression model including Pre-Leu **(A)**, Pre-Neut **(B)**, Pre-Lymph **(C)**, and Pre-NLR **(D)** adjusting for the following covariates: age, gender, tumor location, histological type, setting of surgery, AJCC/UICC TNM stage, and presence of residual tumor. Values in parenthesis are 95% confidence intervals (CI) for HR. Box plots on top of each figure represent the distributions of each parameter.

**Figure 3:**
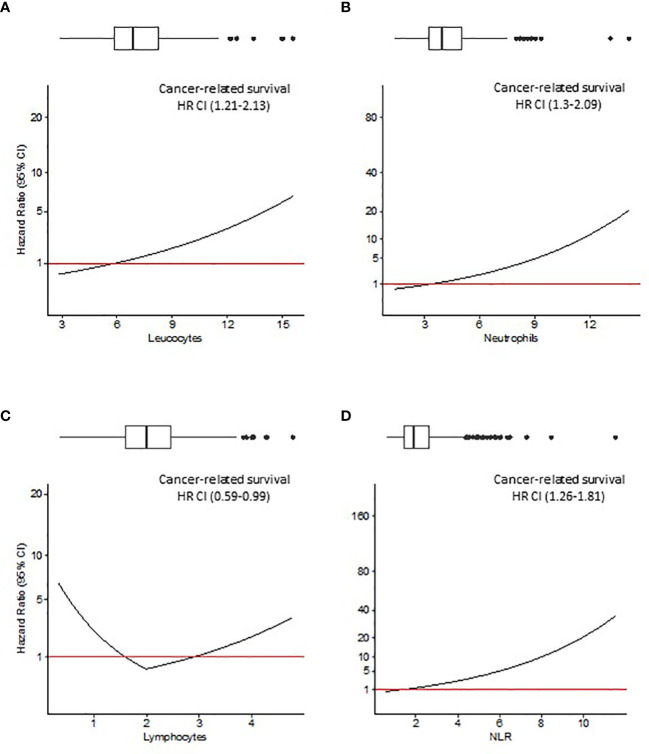
Cancer-related survival (CRS) analysis. Hazard ratio (HR) resulting from Cox regression model including Pre-Leu **(A)**, Pre-Neut **(B)**, Pre-Lymph **(C)**, and Pre-NLR **(D)** adjusting for the following covariates: age, gender, tumor location, histological type, setting of surgery, AJCC/UICC TNM stage, and presence of residual tumor. Values in parenthesis are 95% confidence intervals (CI) for HR. Box plots on top of each figure represent the distributions of each parameter.

Since Pre-Leu, Pre-Neut, Pre-Lymph and Pre-NLR were tested as continuous variables, the hazard ratio (HR, 95% CI) deriving from multivariable analysis was plotted against the values of the parameters. **Figures 2**, **3** show the trend of the HR for OS and CRS, respectively. Both Figures show a progressive rise in the HR with increasing values of Pre-Leu, Pre-Neut, and Pre-NLR. It should be noted how the curve is particularly steep for Pre-NLR as soon as the value exceed the threshold of 3. On the other hand, the HR associated with Pre-Lymph shows a dual trend, consisting of a sharp increase in HR for values of Pre-Lymph below 1.5 x 10^9/L, but also a moderate rise for values above 3 x 10^9/L.

### 3.4 Effect of the interval time before diagnosis

To further evaluate the interaction between pre-diagnosis interval and survival, the interval between Pre-CBC sample and surgery was sub-categorized into 4 groups (24-36 months, 37-60 months, 61-120 months, and >120 months before surgery). Survival curves for CRS were built estimating the survival probability of the patients in the different time intervals and considering the 1^st^ quartile, median and 3^rd^ quartile of Pre-Leu, Pre-Neut, Pre-Lymph and Pre-NLR (**Supplementary Figure 1**). As depicted in the Supplementary Figure, CRS of patients with Pre-Leu, Pre-Neut and Pre-NLR in the 3^rd^ quartile was worse than that of patients with median and 1^st^ quartile values, regardless of the interval between pre-diagnosis sample and surgery. On the contrary, patients with Pre-Lymph values in the lowest (1^st^) quartile showed worse CRS after surgery, compared to the median and 3^rd^ quartile. Interestingly, differences between curves were more pronounced when the Pre-CBC sample was taken between 24 and 36 months before surgery, compared to patients whose Pre-CBC dated over 120 months before diagnosis.

## 4 Discussion

Mounting evidence in recent years advocates a relevant role to chronic inflammation and host immune system in cancer development, progression, and prognosis (8, 9, 28). Notably, the ability to escape immune destruction is now considered within the fourteen core hallmarks of cancer (16). Many studies investigated the relationship between altered immune profile at diagnosis and survival in CRC (17, 18, 29–31) and other solid tumours (12, 15, 32, 33), but none assessed longitudinal changes in these parameters nor the association of Pre-CBC and post-operative survival. In fact, this is the first study ever that investigated the relationship between pre-diagnosis immune profile (at least 24 months before surgery) and cancer survival. The main findings of our study are: i) changes in the immune profile happen years before CRC diagnosis, with a rising trend for Pre-Leu, Pre-Neut and Pre-NLR, and a decreasing trend for Pre-Lymph; ii) Pre-CBC values are independent prognostic factors at multivariable analysis for OS and CRS; iii) the HR for OS and CRS increases proportionally with increasing values of Pre-Leu, Pre-Neut, and Pre-NLR, while the association with Pre-Lymph shows a dual trend; iv) Patients with the highest values of Pre-Leu, Pre-Neut and Pre-NLR or with the lowest values of Pre-Lymph present worse CRS after surgery, and the differences between sub-groups are more pronounced approaching the date of diagnosis.

Despite the association between inflammation, immune system and cancer is a well-studied topic, the body of evidence on pre-diagnosis inflammatory markers and increased risk of cancer is scarce. Some studies from primary care evaluated the rate of incident cancers in subjects with raised inflammatory markers through the analysis of cancer and population-based registries. Although the incidence of cancer was higher in patients with raised pre-diagnosis inflammatory markers, these presented poor sensitivity (25, 34, 35). Zhou and colleagues analyzed primary care blood tests up to 12 months prior to diagnosis in 4533 patients who developed bladder and renal cancer, and found increased rates of raised inflammatory markers from 6-8 months prior to diagnosis (22). Similarly, Moullet and colleagues identified low hemoglobin, high platelets and increased inflammatory markers as early as 9 months prior to the diagnosis of CRC (24). In line with the results of these studies, we also described a progressive change in CBC as early as 24 months prior to diagnosis, with a tendency of Pre-Leu, Pre-Neut, and Pre-NLR to increase, and a decreasing trend for Pre-Lymph.

Interestingly, the analysis of approximately 440000 participants from the large prospective UK Biobank identified a positive association between four immune-related markers (systemic immune-inflammation index [SII], NLR, platelet-to-lymphocyte ration [PLR], and lymphocyte-to-monocyte ratio [LMR]) and risk of several cancers. With respect to CRC, the authors described a positive association with SII, NLR, and PLR, and a negative association with LMR. Also, the association between systemic inflammation markers and the risk varied according to the time between blood drawn and diagnosis. Specifically, no clear association was observed until 4-5 years prior to the diagnosis, with subsequently elevated HR estimates within the last year before diagnosis (23). Apart from Pre-Leu, Pre-Neut, Pre-Lymph, and Pre-NLR, we also considered Pre-LMR as a potential immune-related marker but we did not include it in the final analysis as Pre-monocyte was available only for 257 patients. However, also in our cohort Pre-LMR showed a negative correlation with survival (data not shown). While the study on the UK Biobank only assessed the correlation of inflammatory markers with the risk of cancer development and did not infer on cancer prognosis, it still highlighted a significant role of blood cell ratios as early biomarkers of immune response shift to an already developing tumor process. The early identification of immunological changes may represent a window of opportunity for early diagnosis of cancer. However, it should be more extensively investigated whether these alterations in Pre-CBC represent the *primum movens* of cancer pathogenesis in predisposed patients or just an epiphenomenon of tumour growth.

The balance between immune surveillance by the cellular immunity and the capability of tumour cells to avoid immune destruction represents one of the crucial points in cancer progression (16). Lymphocytes can act as tumour suppressors through the release of lytic components and direct cell-cell interaction (36, 37). Neutrophils, on the other hand, can prompt cell proliferation because of inflammation and production of cytokines and chemokines that promote tumour growth, angiogenesis, and metastasis (36, 38, 39). Accordingly, increased NLR represents an easily available and adequate biomarker of a pro-tumorigenic immune shift. It should be noted that the median values of Pre-CBC components were within normal ranges. Therefore, although we identified a significant prognostic role of these immunological parameters, further studies on specific sub-populations will be needed to aid clinical applicability.

Some limitations of the study should be acknowledged. Due to its retrospective nature, it was not possible to retrieve complete data for all variables, in particular Pre-CBC for all patients. From an initial population of 1674 patients, we were in fact able to obtain Pre-CBC just for 334 patients, who were however representative of the global cohort as demonstrated by clinical and pathological characteristics (**Table 1**). Also, although we excluded patients with clear evidence of an ongoing infection, autoimmune disorders, treated with corticosteroids or immunosuppressive/immunomodulatory therapy, and blood samples associated with admission to the Emergency Department, it may be possible that some alterations in Pre-CBC were consequence of a subclinical inflammatory or infective condition. Finally, no information is available on the different subtypes of lymphocytes. It is sensible to believe that the involvement of different subpopulations may account for the dual trend in HR in relationship to Pre-Lymph values.

On the other hand, it should be noted that this is the first study evaluating pre-diagnosis CBC and its correlation with post-operative survival in a fairly large cohort of surgically resected CRC. Further studies will be required to confirm our results, as well as to deeply investigate the complex interaction between cancer and immunity cells. Future perspective may involve characterisation of the role of the different sub-classes of lymphocytes and neutrophils together with the cross-talk between circulating and TME cells.

## 5 Conclusions

Given the known correlation between inflammation and cancer, and the prognostic role of some inflammatory markers, our study aimed at assessing changes in complete blood count occurring even before the diagnosis of CRC. After controlling for relevant covariates, Pre-Leu, Pre-Neut, Pre-Lymph, and Pre-NLR proved to be independent prognostic factors for OS and CRS.

## Data availability statement

The raw data supporting the conclusions of this article will be made available by the authors, upon reasonable request to the corresponding author.

## Ethics statement

The studies involving human participants were reviewed and approved by Comitato Etico per la Sperimentazione Clinica delle Province di Verona e Rovigo, Azienda Ospedaliera Universitaria Integrata Verona. The patients/participants provided their written informed consent to participate in this study.

## Author contributions

Conceptualization: GT, SU, VBr, and CP Methodology: GT, SC, CP. Software: SC. Validation: CC, SZ. Formal analysis: GT, SC. Data curation: GT, CC, SZ. Writing—original draft preparation: GT, SC, SU, CP. Writing—review and editing: GT, CC, VBa, AS, GL, AR, VBr, AG, CP. Supervision: SU, VBa, GL, AS, AR, VBr, AG, CP. All authors have read and agreed to the published version of the manuscript.
