# Supplementary material for: Pre-diagnostic prognostic value of leukocytes count and neutrophil-to-lymphocyte ratio in patients who develop colorectal cancer
Source: Front Oncol. 2023 Jun 5;13:1148197. doi: 10.3389/fonc.2023.1148197 (PMC10277676; doi:10.3389/fonc.2023.1148197)
Supplement: Supplementary file 1 [file DataSheet_1.docx]

Supplementary Material

Pre-diagnostic values of leukocytes count and neutrophil-to-lymphocyte ratio in patients who develop colorectal cancer

Giulia Turri^1^, Simone Caligola^2^, Stefano Ugel^2^, Cristian Conti^1^, Silvia Zenuni^1^, Valeria Barresi^3^, Andrea Ruzzenente^1^, Giuseppe Lippi^4^, Aldo Scarpa^3^, Alfredo Guglielmi^1^, Corrado Pedrazzani^1*^

*** Correspondence:** Prof. Corrado Pedrazzani, Division of General and Hepatobiliary Surgery, Department of Surgical Sciences, Dentistry, Gynecology and Pediatrics, Verona University Hospital, Piazzale L. Scuro 10, 37134 Verona, Italy. Tel: ++39 (0)45 8126719; Fax: ++39 (0)45 8027426; E-mail: [corrado.pedrazzani@univr.it](mailto:corrado.pedrazzani@univr.it)


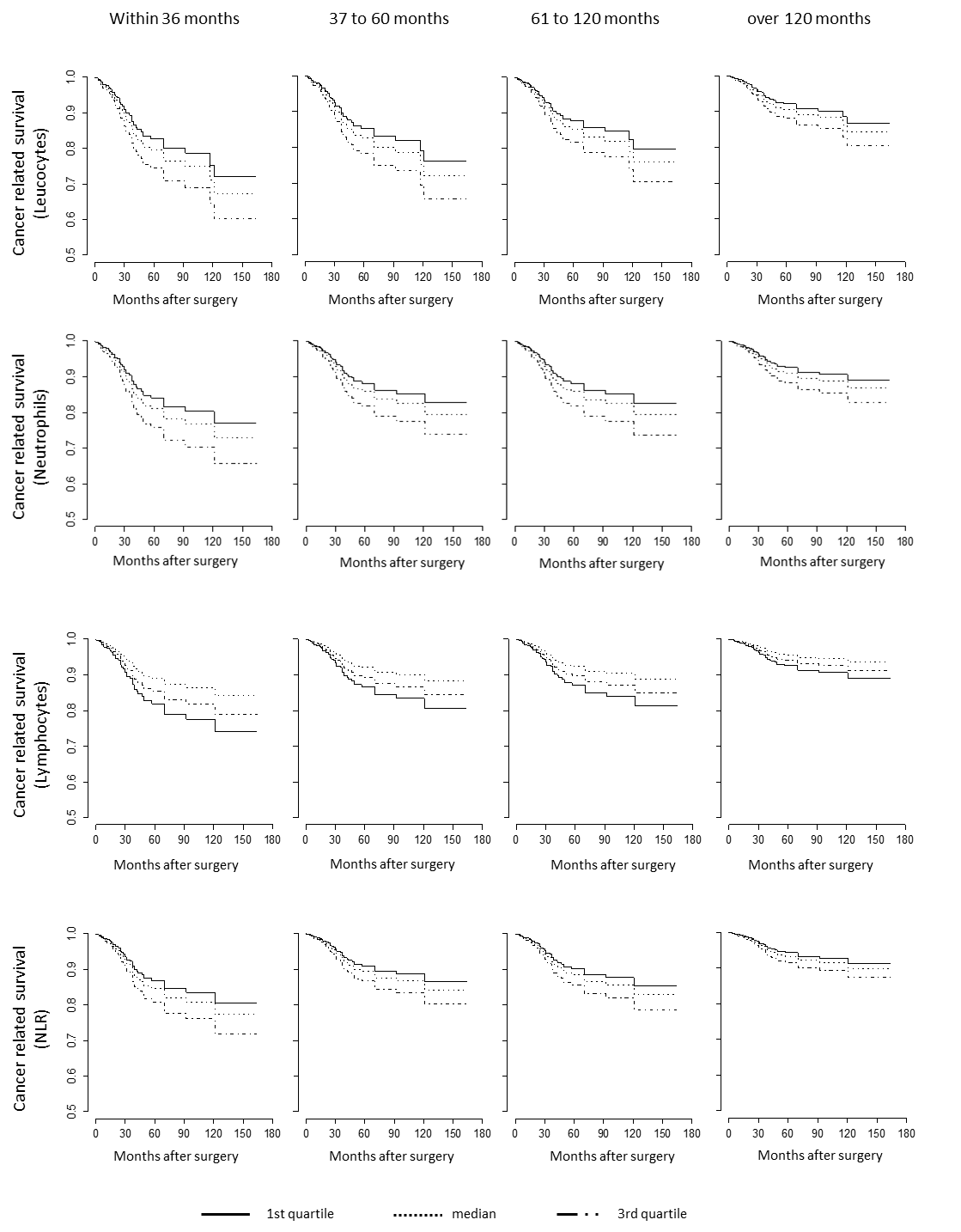


**Supplementary Figure 1.** Cancer-related survival (CRS) analysis. Survival probability resulting from Cox regression model including (A) Pre-Leu, (B) Pre-Neut, (C) Pre-Lymph, and (D) Pre-NLR in 4 intervals between Pre-CBC and surgery (24-36 months, 37-60 months, 61-120 months, and >120 months before surgery). The model was adjusted for the following covariates: age, gender, tumor location, histological type, setting of surgery, AJCC/UICC TNM stage, and presence of residual tumor.
